# Who Talks About Flood Risks and Climate Change Adaptation? Analysis of Social Interactions in Three Countries

**DOI:** 10.1111/risa.70213

**Published:** 2026-03-02

**Authors:** Thorid Wagenblast, Amineh Ghorbani, Martijn Warnier, Tatiana Filatova

**Affiliations:** ^1^ Department of Multi‐Actor Systems, Faculty of Technology, Policy and Management Delft University of Technology Delft the Netherlands

**Keywords:** behavior, diffusion, flood adaptation, household survey, social amplification, social norm

## Abstract

People's risk perceptions are crucial for climate change adaptation, influencing individual decisions and policy effectiveness. Although many studies highlight the importance of social influences and social norms in this context, the mechanisms through which they shape individual risk perceptions and adaptation behavior remain unclear. To address this gap, we analyze cross‐country survey data (*N* = 1612) from coastal areas in the Netherlands, United Kingdom, and the USA with a focus on flood risk and adaptation behavior. Our statistical analysis reveals several important patterns in social interactions, and the ways in which these social interactions influence individual risk perceptions. First, we find limited social engagement regarding risks and adaptation, with a significant portion of respondents (50%) reporting no interactions with peers on these topics. Among those who do engage, social interactions on flood risk and adaptation appear infrequent (fewer than five times per year). Second, contrary to common assumptions, individuals who discuss flood risk and adaptation, rarely do so with neighbors. Moreover, homophily—shared socio‐demographic characteristics—is not the primary determinant of who interacts on the topic. Third, we see that those with hazard experience and those with higher risk perceptions are more likely to interact with others on the topics of these risks and climate adaptation, confirming that social amplifications might be in place. These findings provide unique insights into the social dynamics underlying the evolution of individual risk perceptions, offering the potential to refine models of social influence in climate change and social tipping points. They also highlight potential synergies between communication strategies and policy tools to support timely and, possibly transformational, adaptation.

## 1 Introduction

People rarely make decisions in isolation. Social influence and individual risk perceptions go hand in hand, especially for decisions involving uncertainty and potentially high costs. This is particularly relevant in the domain of climate change adaptation. Adaptation is needed across scales: Besides public efforts by governments, increasingly, people must also adapt to climate‐induced extreme events. Floods (pluvial, fluvial, and coastal) and storms are the most widespread and costly hazards, and climate change makes them more frequent and intense (IPCC 2021). Currently, the pace at which adaptation is happening cannot keep up with the increasing magnitude of these events (UNEP 2024). Furthermore, adaptation often occurs in incremental steps, despite the growing need for transformational approaches (Kates et al. 2012). One way to achieve transformational adaptation is through the diffusion and mass uptake of individual measures: Social influences can amplify individual behavioral changes, triggering social tipping processes (Castilla‐Rho et al. 2017) and leading to transformational adaptation (Wiedermann et al. 2020; Wilson et al. 2020).

Social influence is a combination of social norms (relatively stable) and social interactions (variable), and plays an important role in shaping individual risk perceptions and adaptation diffusion. Theoretical research conceptualizing this link includes the Social Amplification of Risk framework (SARF) that focuses on how social processes, including informal interactions and experiences, amplify risk perception (Binder et al. 2011; Brenkert‐Smith et al. 2013; Kasperson et al. 2022). Other relevant theoretical work includes the Consumat framework, which conceptualizes individual human behavior contingent on social influence if uncertainty of decision consequences if high (Jager and Janssen 2012), or the concept of social capital with links to individual adaptive capacity and risk perception (Adger 2003; Bixler et al. 2021). The theoretical research has been confirmed by empirical evidence showing that social influence and norms have a significant effect on flood risk perception and adaptation decisions (Bubeck et al. 2012; Van Duinen et al. 2015; van Valkengoed and Steg 2019; Noll et al. 2022b). Simulation studies on climate adaptation uptake and diffusion assume that social influence impacts individual risk perceptions and adaptation decisions (Kremmydas et al. 2018; Taberna et al. 2020). Specifically, individuals are influenced in their perceptions of risks, effectiveness, and coping strategies, which, in turn, shape household adaptation decisions (see Kuhlicke et al. (2023) for an overview). In particular, agent‐based modeling has pioneered in explicitly testing the role of social influence on individual adaptation decisions and overall risk reduction (e.g., Haer et al. 2016; Van Duinen et al. 2016; Abebe et al. 2020; Choquette‐Levy et al. 2021). Yet, even empirical simulations that capture dynamic social influences still rely on stylized assumptions related to network structure, who influences whom, and on which information dimensions. In turn, empirical data collection via social surveys—usually a snapshot in time—rarely go beyond simply detecting that currently‐prevailing (static) social norms affect individual adaptation decisions. Nevertheless, who interacts with whom, how often people engage in social interactions, and which information they exchange—about probabilities, damages, or coping strategies—significantly impacts individual perceptions, adaptation uptake, and residual damages (Wagenblast et al. 2024). Yet, the absence of empirical evidence on how social influence affects individual risk perceptions and private adaptation decisions, scholars, and practitioners must rely on either a static picture of the role of the past snapshot of social norms or empirically‐ungrounded stylized dynamic simulations of social influence. Both might misguide the anticipated scope and scale of individual adaptation uptake and misinform the design of effective risk reduction policies.

Furthermore, social influence plays an important role when searching for policies to support individual adaptation or political behavior and the acceptability of public adaptation (Bimber and Gil De Zúñiga 2022). This is true especially when we think about information policies that are often used to inform individuals to trigger them to act (e.g., publicly available flood maps to inform households of the flood risk or government programs for subsidies for flood‐proofing homes (Huang and Lubell 2022)). When we neglect the social dynamics between individuals, these can easily backfire, or the effect might be diluted. Effective information policies could accelerate or steer adaptation processes. Consequently, the understanding of the nature of social influence in the climate risks and adaptation context—that is, who communicates with whom, how often, and about which element of risk—is crucial to carrying out climate change adaptation effectively. Social influence is also important to speed up adaptation and to scale it up to the level of transformational change. If we want to enable triggers of “positive” change that amplify individual behavioral changes, like the upscaling of private adaptation via social tipping, the underlying social influences need to be understood. Getting empirical insights on the role of social influence on individual risk perceptions and adaptation behavior is one of the levers toward a more adapted and climate‐resilient world.

Our article seeks to address these research gaps by revealing what form social influences currently take in the context of climate‐induced risks and adaptation, particularly flooding, and how this relates to widespread assumptions on how social influence spreads between individuals in this context. Towards this end, we aim to provide insights on the following research questions: What is the intensity and the exact thematic focus of the social interactions about flood risks and adaptation? (RQ1); Does the intensity of social interactions on this topic correspond to differences in individual risk perceptions and socio‐demographic characteristics? (RQ2); What are the key shared characteristics of participants in the peer discussions focused on climate risks and flood adaptation? (RQ3). Using original survey data (*N* = 1612) from three countries—the Netherlands, United Kingdom, and the USA—we address these research questions, focusing on floods as the exemplar most costly and widespread climate‐induced hazard. The article proceeds as follows. First, the Background and Methods section presents our hypotheses, our survey data, and the data analysis methods. Then we address the research questions in the Results, followed by a Discussion on the hypotheses, implications and further work, and the Conclusions.

## 2 Background and Methods

### 2.1 Theoretical Background, Hypotheses, and Propositions

Social influence can come in various forms. It has to do with individuals and how their worldview is shaped by perceived social norms and by information received from others. Lim (2022) defines “social influence” as the way in which society impacts individuals in their values, beliefs, perceptions, attitudes, intentions, and behaviors, where the individual and society are connected via social interactions. This focus on social interactions also appears in other literature on social influence. For example, Flache et al. (2017) define “social influence” as the modification of opinions, attitudes, beliefs, and/or behavior towards those whom they interact with. Others state that “social influence” is the relationship between two entities that interact, the influencer and the influenced, whose relationship is not necessarily symmetric (Peng et al. 2018). Generic across these definitions is that social interactions play an important role in shaping social influence. Social interactions constitute the exchange of information between individuals about what is on their minds. This triggers an alignment process happening over time and across space (Gallotti et al. 2017). For this study, we look at social interaction as the means through which social influence—the dynamic adjustment of perceptions or update of information about risks and individual adaptation measures—occurs.

**FIGURE 1:**
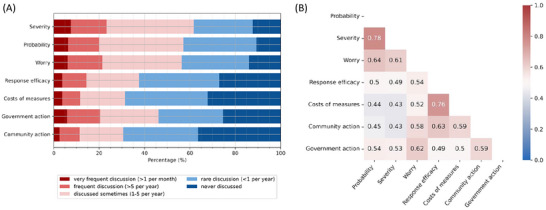
Frequency of engaging in social interactions on the topics related to flood risks and climate change adaptation. (A) The bar plot reports the analysis of the subset of the respondents who reported discussing any of these topics (*N* = 481). (B) Correlation between the discussion frequency of the different interaction topics. Here, Probability reports how frequently an individual discusses the topic of probability of a climate‐induced hazard with peers. Respectively: Severity—potential severity of a flood event; Worry—how much they worry about a potential flood event; Response efficacy—effectiveness of different adaptation measures; Costs of measures—costs of different adaptation measures; Community action—what actions they can take as a community to adapt; Government action—government's adaptation actions or lack of those.

Various empirical studies show that social influence in different forms plays a role in households’ decisions to take climate adaptation or protective measures. This is often measured through perceived injunctive, descriptive, or subjective social norms (e.g., Bubeck et al. 2013; Lo 2013; Ridzuan et al. 2024) or as social capital (Bixler et al. 2021). The effect of these empirically elicited social norms/interactions is often as (or more) important than rational judgments about probabilities and damages or costs and effectiveness of measures (see van Valkengoed and Steg ((2019) for a meta‐analysis). Despite this strong empirical evidence on social interactions and norms affecting individual risk perceptions and adaptation decisions, rarely did empirical analysis go beyond simply detecting this link or capturing social norms as a static snapshot. Given the lack of detailed data, social interactions are rarely considered explicitly in empirical studies in the context of climate change. Scarce evidence suggests that people do not care enough about climate change to discuss it often with each other (Ballew et al. 2019; Leiserowitz 2022). For the purpose of this study and in the context of flood risk, adaptation, and related topics, we consider discussion frequencies of, on average, less than once a year as “infrequent”, more than 5 times a year as frequent (see Figure 1, Appendix B for more information).

***Proposition P1***. People engage in discussions about climate‐induced risks, like flooding, and strategies to adapt to them infrequently (less than once a year on average).

The decision to take (individual) adaptation measures is driven by the perceptions of climate hazards and the ability to respond effectively (see reviews by Kuhlicke et al. (2020), van Valkengoed and Steg (2019)(). These perceptions are heavily shaped by environmental cues, the concerns expressed within one's social network, the observed actions of others (i.e., descriptive social norm), and perceived expectations of socially approved actions in a risky situation (i.e., injunctive social norm). Theories of individual decision‐making and behavior, such as Protection Motivation Theory (PMT), the Model of Action Phases (MAP), and Protective Action Decision Making (PADM), emphasize the importance of perceptions related to both threat and the ability to act, often distinguishing these as separate, yet interlinked, components of the decision‐making process (Kuhlicke et al. 2020). Whether social influence affects threat appraisal and coping appraisal remains underexplored. This leads us to the next aspect of our exploration.

When climate risks are discussed, the concepts constituting threat appraisal are discussed together, as indicated by their discussion frequencies. The same holds for the coping appraisal.

Empirical research has shown that higher risk perception and past hazard‐related experience, such as flood experience, increase the intentions and behavior of taking adaptation measures (Pozzi and Hillis 2023; Terpstra and Lindell 2013). Therefore, higher risk perceptions and hazard experience are also likely to increase the likelihood of social interactions on the topic. This aligns well with SARF that develops theoretical foundations for linking social interactions (interpersonal communication) with the amplification of risk (Kasperson et al. 1988, 2022). Using this framework, Binder et al. (2011) find that for biological risks, the discussion frequency is positively related to risk perception. For wildfire risks, Brenkert‐Smith et al. (2013) expand the SARF to include social processes like information sources and social interactions—similarly, they find that both formal and informal risk‐related interactions increase the perceived risk. This leaves a situation where some audiences require amplification, while others an attenuation of risk (Dow and Tuler 2022). With this in mind, we formulate the following hypothesis.

Individuals who perceive higher risks are more likely to engage in communication on the topic.

There is limited knowledge regarding the nature of social interactions in the context of individual climate adaptation, especially regarding the structure of social networks through which specific information spreads. For example, we know little about how many and what type of individuals discuss climate‐related risks, with whom, how often, and how dense such discussion networks are. In other words, science knows little about how social interactions in the context of climate risks and adaptation might evolve over time. Yet, simulation studies demonstrate that the structure and intensity of interactions are decisive for how quickly behavior is adopted (Centola and Macy 2007; Niamir et al. 2020). Looking at studies of social influence, homophily is often mentioned as the core assumption regarding who individuals interact with (Flache et al. 2017). The assumption is that individuals who are similar to one another are more likely to connect with each other and talk (McPherson et al. 2001). Such peer networks are relatively homogeneous with respect to sociodemographic, behavioral, and intrapersonal characteristics. Though homophily is multi‐dimensional and can include opinions or behaviors, the strongest divides come on the dimensions of race and ethnicity, followed by age, religion, education, occupation, and gender (McPherson et al. 2001). These homophily networks to which an individual connects might be important, especially to generate a critical mass and start social movements regarding more complex issues (Guilbeault et al. 2018). Our analysis centers on socio‐demographic forms of homophily, given their prominence in the literature, the strength of the divisions they reflect, and the relative ease of observation.

People interact with those who are like them in terms of socio‐demographic characteristics.

Furthermore, neighbors are typically considered an important peer group when it comes to decisions that concern people's homes. For example, in the household energy behavior literature, peer groups based on spatial proximity and neighborhood effects play a significant role (Wolske et al. 2020); the same is true for mortgage refinancing decisions (McCartney and Shah 2022) or farmers’ adoption of new technologies (Case 1992). Especially when it comes to climate change and adaptation to its adverse effects, households might look to their neighbors for cues and guidance. Though little empirical evidence exists on whether this is true, this assumption is often supported by the arguments that due to the spatial proximity, neighbors often share a similar risk profile and living conditions. This leads to our next hypothesis.

People interact with their neighbors to inform their decisions to take individual property‐level adaptation measures.

### 2.2 Survey Methodology and Key Variables

**FIGURE B1 risa70213-fig-0003:**
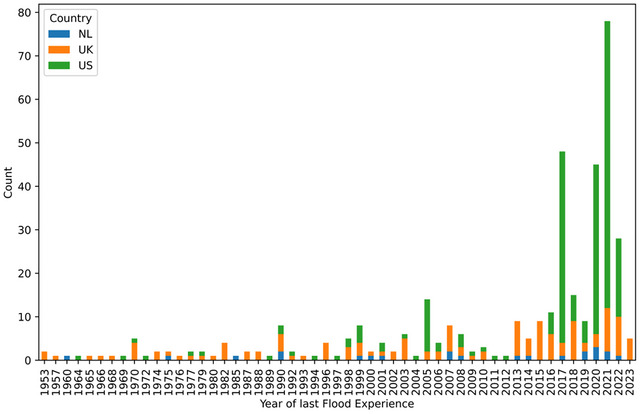
Year of last flood experience for those respondents with flood experience.

In July–August 2023, we conducted a cross‐sectional household survey in flood‐prone coastal areas in three countries to collect primary data on how people influence and are influenced in their flood risk perception and adaptation decisions. To reach out to the respondents in different countries, we relied on the online surveys using the help of YouGov^1^. The respondents of the current survey (*N* = 1612) are situated in the United States (Miami, Houston, New Orleans), United Kingdom (Norfolk and Suffolk coast, Somerset, London), and the Netherlands (South Holland). These study areas are selected because they are vulnerable to coastal, river, and flash floods and to sea level rise, and will have to deal with increasing flood risk. This does not necessarily mean that respondents live in official flood zones as these are often based on past hazards, might omit particular forms of flooding like heavy rainfall (Highfield et al. 2013) and, therefore, might not be able to represent the current or future risk adequately. While this does not mean that every household has experienced or will experience a flood‐related extreme weather event in the coming years (see Figure B1 in Appendix B for when respondents last experienced a flood), the risks and, therefore, awareness in the regions as a whole are expected to increase (Magnan et al. 2023). Furthermore, our selected locations represent different social, institutional, and geographic contexts with government involvement ranging from taking a strong central role in protection (Netherlands) to more individual responsibility to adapt (United States).

The survey is part of our longitudinal study conducted in the context of the ERC SCALAR project (Filatova et al. 2022). While the first four waves focused extensively on household flood adaptation (Noll et al. 2022a, 2022b), this fifth wave has a specific focus on social influence in that context. YouGov supported us with representative panels of respondents that match national statistics and obtained informed consent. They ensure quality through various measures like blind selection from the panel pool and inviting participants before announcing the topic to avoid self‐selection bias, verifying personal details, and excluding speeding respondents (clicking too rapidly to allow reading) and respondents who consistently click the same option (e.g., always the first answer). Furthermore, the surveys are accessible online and via mobile phones to reduce reliance on the internet at home. As this survey is part of a longitudinal study, part of our respondents are the participants from the four earlier waves contingent on the retention rate in each country. In addition, in the Netherlands and the United Kingdom, new respondents were recruited. The socio‐demographic characteristics of our survey samples and their comparison to the representative population of the country or regional population characteristics are provided in Appendix A. The respondents in general are slightly older, have a higher income and are better educated than the general population. The difference is most noticeable in the United States and could be related to the retention of respondents there.

To design the survey questionnaire with a focus on social influence, we reviewed literature that used surveys to study social interactions, norms, and influence in various contexts (Buzelli et al. 2022; Mouter et al. 2022; Schwenk 2009; Stedman 2004; Van Duinen 2015; Van Duinen et al. 2015; Vignola et al. 2013). Consequently, we selected the questions eliciting the nature of social interactions, frequency, and the subject of information exchange and adapted them to the context of climate risks and private property‐level adaptation (see Appendix B for the survey questions used in this analysis and our open‐access full questionnaire on DANS (Filatova et al. 2022)). We have developed the survey in English, and YouGov helped us translate it into the respective languages of each country. The translations were reviewed by independent native speakers from each of the case studies’ countries, namely the local field teams of YouGov and scientists in our network.

Next to the socio‐demographic data (age, income, education, gender), we compile data on social interactions (Appendix B). We ask respondents about the number of conversations they engaged in on the topic of flood risk and climate change adaptation and about how many people they had talked to on this topic within the last year. If respondents said that they had at least one discussion on the topic of flooding, climate change adaptation or sea‐level rise in the last year, we asked about the discussion frequency on the following issues: “(i) How often floods could affect (or already affect) the area [they] live in; (ii) how devastating floods could be or are in [their] area; (iii) how worried [they] and/or [their peers] are about floods; (iv) possible effectiveness of various flood adaptation measures that people can implement on own home; (v) possible costs of various flood adaptation measures that people can implement on own home; (vi) how [they] could take adaptation measures as a group in [their] neighborhood or community; (vii) the government's plans or lack of them to deal with flooding in [their] area.” Furthermore, data on the risk perception (perceived probability of experiencing a flood where they live, worry about potential flooding, potential severity/damage in case of flooding at their place) and flood experience of the respondents were collected.

We used the name generator (NG) approach (Marsden 1987; Merluzzi and Burt 2013) to get specific insights into the characteristics of peers with whom our survey respondents engage in conversations regarding flood risks and climate adaptation. The goal is to understand what kind of people interact with each other while respecting respondents’ privacy. The NG approach is a common way to get information on network characteristics and structure, including information on the (socio‐demographic) characteristics of each alter and the relationship between respondents and their peers (Marin and Hampton 2007). With this approach, people (ego) are asked to give names, in this case, of up to 3 people (alters) outside their household with whom they discuss concerns about and possible preparations for flooding. While respondents are asked to think about real people in their network, they are offered a possibility to provide fictitious names to label these contacts to ensure anonymity for both respondents (ego) and the people behind the given names (alters). They then proceed to answer questions specifically about interactions with that person with Name X. This includes information on their nature of relation (family, friends, neighbors, …), their similarity (income, education, age, flood exposure), geographical distance, discussion frequency in general and on the topic, and their experience in terms of property‐level climate adaptation (see Appendix B for the questions used for this analysis).

We removed 5 outliers from the dataset due to unrealistically high reported interaction numbers (>100 peers), leaving us with data from 1607 respondents (UK: 741, NL: 418, US: 448). Table 1 lists the variables we use to study the hypotheses related to our research questions; Appendix B provides further details on the corresponding survey questions and descriptive statistics.

**TABLE 1:** Guiding questions, hypotheses, and variables used for each. A full description of the variable names, the corresponding questions, and answer options can be found in Appendix B.

Research question	Related hypotheses/ propositions	Corresponding variable names	Methodology
What is the intensity and the exact thematic focus of the social interactions about flood risks and adaptation? (RQ1)	P1. *People engage in discussions about climate‐induced risks, like flooding, and strategies to adapt to them infrequently (less than once a year on average)*. *H1. When climate risks are discussed, the concepts constituting threat appraisal are discussed together, as indicated by their discussion frequencies. The same holds for the coping appraisal*.	Discussion frequency probability Discussion frequency severity Discussion frequency worry Discussion frequency response efficacy Discussion frequency costs of measures Discussion frequency community action Discussion frequency government action	Descriptive analysis (P1) and Correlation analysis (H1)
Does the intensity of social interactions on this topic correspond to differences in individual risk perception and socio‐demographic characteristics? (RQ2)	*H2. Individuals, who perceive higher risks, are more likely to engage in communication on the topic*.	Gender (fraction male) Age Education Income Fraction with flood experience Worry Perceived probability Number of flood conversations Number of people talked to Number of names given in NG	Descriptive analysis, K‐median clustering combined with descriptive analysis per cluster
What are the key shared characteristics of participants in the peer discussions focused on climate risks and flood adaptation? (RQ3)	*H3a. People interact with those who are like them in terms of socio‐demographic characteristics*. *H3b. People interact with their neighbors to inform their decisions to take individual property‐level adaptation measures*.	Gender Age Education Income House type Flood experience Worry Perceived probability Who Similarity	Hierarchical clustering on two dimensions and overlapping the results

### 2.3 Analysis Methods

To answer the first research question, we use descriptive analysis to determine the general discussion frequency of various topics related to climate risk and private adaptation (Table 1), as knowing the number of people participating in interactions on specific topics has direct impacts on social network construction and information diffusion (Dunbar 2011; Flache et al. 2017). Furthermore, we look at the correlations between the discussion frequencies of the different topics. The underlying assumption is that if respondents report similar discussion frequency for different topics, resulting in a higher correlation among them, they probably discuss them together.

In addressing the second research question, we use three different variables to operationalize social interactions and examine their relationships with individual risk perception, namely: (i) The number of social interactions a person engaged in the last year regarding climate‐induced risks (here floods and sea level rise) and adaptation (*number of flood conversations*), (ii) the number of peers with whom a respondent engaged in conversations regarding climate‐induced risks and adaptation in the last year (*number of people talked to*), and (iii) the number of names reported in the NG section (*number of names given in NG*, a maximum of 3 could be given). We first split the survey population into those who interact on this topic (*Communicators*) and those who do not (*Non‐Communicators*). This is done based on whether they filled the NG (*number of names given in NG* = 0 vs. *number of names given in NG* > 0). We then check their characteristics in terms of social interactions, risk perception, and socio‐demographics. We further want to gain additional insights into the *Communicators* group: We cluster over the three dimensions of social interactions the proportion of the survey population who reported engaging in conversations with peers on the topic of climate risks and adaptation. The goal is to separate the *Communicators* into different levels of social interactions. As the data is not normally distributed and contains outliers, we use a k‐median clustering algorithm with Manhattan distance metric (Park and Jun 2009). We select the number of clusters using the Davies‐Bouldin index (Davies and Bouldin 1979), check the interpretability of the data within the different clusters, and then link them to individual risk perceptions and socio‐demographics.

To address the third research question on the characteristics of people participating in discussions on flooding and climate change adaptation, we used a subset of the questions posed in the NG approach. Specifically, we analyzed the answers given to the question: “Who is this person to you?” Respondents could respond with “yes” or “no” for each Family/Friend, Colleague, Neighbor, Online Acquaintance, Government Representative, or Other, with multiple “yes” or only “no” answers possible. For the similarity, the question was, “Do you consider yourself to be similar to each of those people in terms of the following characteristics…?” Income, Age, Level of Education, Flood Exposure, and House Type. Again, multiple yeses or all‐no answers were possible. As the data is binary (yes/no), and we want to identify homogeneous clusters, we use hierarchical Ward clustering with Euclidean distance metric (Ward 1963). We apply this to the responses from the two questions separately and decide on the number of clusters displayed based on the interpretability of the cluster's composition. This means we select the number of clusters based on roughly similar size and height in the dendrogram, what we find in them and whether this relates to meaningful groups. Eventually, we overlap the different clusters for the answers to the two questions to better understand the relation between relationship and similarity.

## 3 Results

We present our data analyses along the stated hypotheses. First, we present the discussion frequencies of different topics surrounding individual flood risk perception and private adaptation, and the correlations among them in Section 3.1. Then, the results related to those who communicate and those who do not are shown in Section 3.2. This includes the difference between *Communicators* and *Non‐Communicators*, as well as the results from clustering into different levels of intensity of social interactions. Finally, Section 3.3 focuses on the social interactions of the Communicators, aiming to shed light on what relationship they have with each other and what their characteristics are.

### 3.1 Discussion Frequency and Topics

Overall, the interaction frequency, specifically on flood risk and private adaptation, is low; discussions on these topics happen on average less than once a year (Figure 1A). Less than 1/3 of respondents (481 respondents out of 1607) gave insights into the discussion frequency of different topics overall; the rest said they had not had any conversation in the last year on any of the topics. The discussions surrounding risk—including the conversations on the likelihood of a flooding event, the potential severity, and the worry about it—happen more often than those on adaptation measures. More than a quarter of the respondents reported that they never discuss response efficacy and costs of potential private adaptation measures, as well as community and government action.

Some patterns emerge after looking at the correlations between the frequency of discussions on different topics (Figure 1B). The correlation between the discussion frequency of the potential severity of a flood event (*Severity*) and the perceived probability of it (*Probability*) is high (*r* = 0.78, *p* < 0.001, *N* = 481). The same is true for the correlation between the costs of potential adaptation measures (*Costs of measures*) and the response efficacy (*Response efficacy*), in other words, the effectiveness of different adaptation measures (*r* = 0.76, *p* < 0.001, *N* = 481). There are also some differences between the countries (see Appendix C for details). Especially for the Dutch data, the correlation in discussion frequency of worry (*Worry*) and government actions (*Government action*), and government and community actions (*Community action*) is high (*r* ≥ 0.74, compared to *r* < 0.6 for the United Kingdom and United States).

These findings confirm our first proposition: Topics surrounding climate change adaptation, specifically adaptation against flooding and SLR, are discussed less than once a year on average, making it an infrequent interaction theme (P1). Furthermore, the high correlations between the discussion of coping factors (*Costs of measures, Response efficacy*), as well as the high correlation between threat factors (*Severity, Probability, Worry*) confirm hypothesis H1.

### 3.2 Characteristics of People Engaging in Social Interactions

#### 3.2.1 Communicators and Non‐Communicators

**FIGURE 2:**
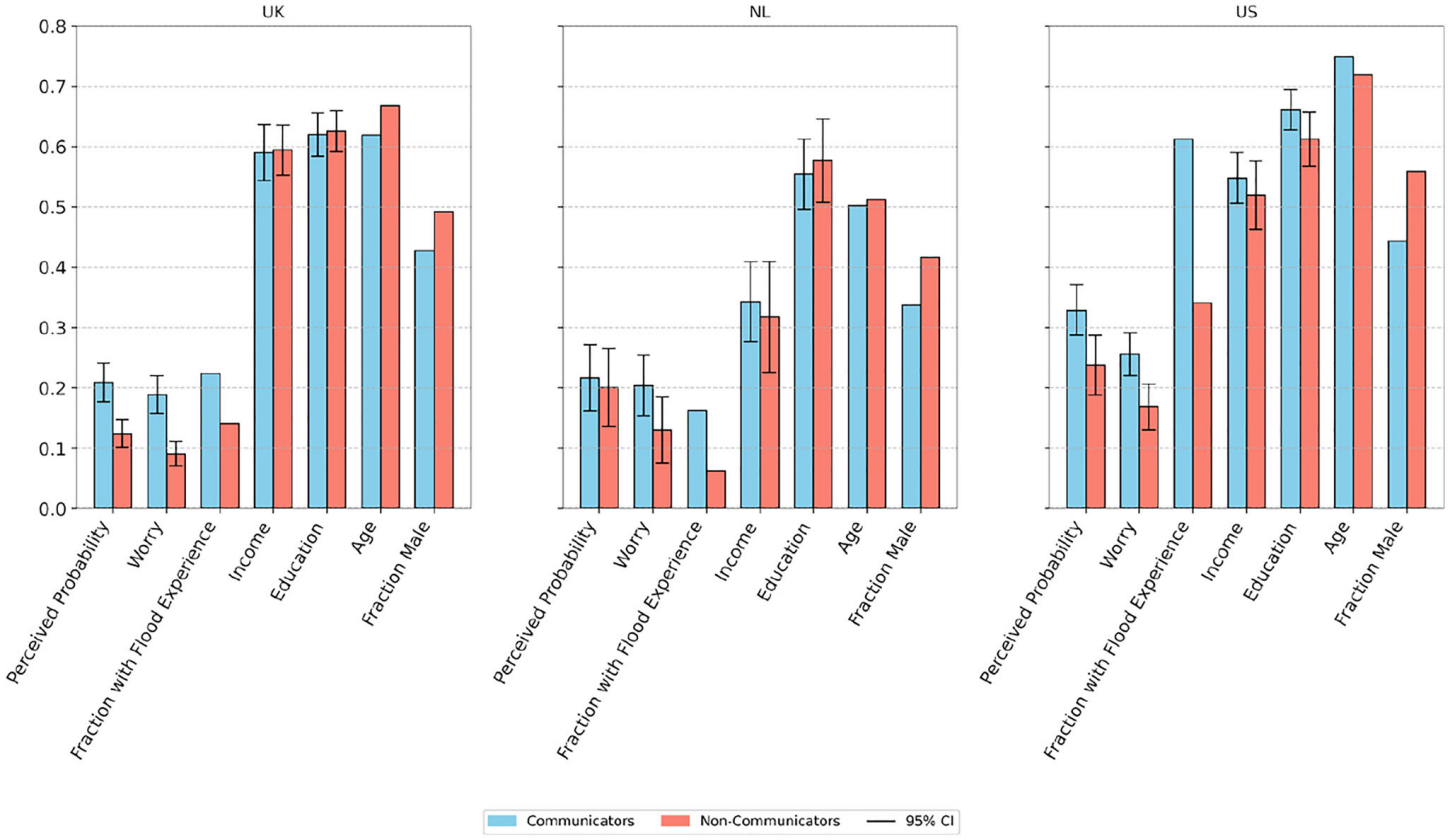
Differences between Communicators and Non‐Communicators in the United Kingdom (UK, *N* = 547), the Netherlands (NL, *N* = 134), and the United States (US, *N* = 343). The histogram bars represent the mean value of selected variables scaled to 0–1 between each variable's minimum and maximum values. Specifically: Perceived probability scale varies between [0–1]; Worry—[not at all worried—very worried]; Fraction of population with Flood Experience—[0–1]; Income—[country‐specific lowest income quintile—highest]; Education—[no school diploma—post‐graduate degree]; Age—[16–24 years of age–65+ years of age]; Fraction of Male—[0–1]. Confidence intervals are removed for Age (age was gathered in six age groups for the US data; for comparison it was dichotomized for UK and NL data).

*Communicators* are those individuals who engage in social interactions over climate‐induced risk (here, flood or sea level rise) and adaptation to it, as measured by the number of conversations they engage with peers on the topic, as well as the number of people they talk to. We denote respondents who reported no such social interactions as *Non‐Communicators*. *Communicators* are significantly different from *Non‐Communicators* on a number of behavioral and demographic characteristics (Figure 2).

Looking at the risk, we see that the proportion of individuals with flood experience is higher among the *Communicators* group. Generally, many US respondents report having flood experience (>60% of those communicating, ∼35% of those who do not). Conversely, among the UK and the NL respondents, the proportion remains below a quarter (*Fraction with Flood Experience*, Figure 2), regardless of whether they report engaging in social interactions or not. Furthermore, the *Communicators* are also more worried about flood risk and climate change (all countries, *Worry*, Figure 2) and have a higher perceived probability of hazard (UK and US, *Perceived Probability*, Figure 2) compared to *Non‐Communicators*.

Considering the demographics, the differences in age, education, and income are small. The UK *Communicators* are slightly older than *Non‐Communicators*, whereas in the United States, they are slightly younger (*Age*, Figure 2). We observe a consistently higher fraction of males among Non‐Communicators (*Fraction Male*, Figure 2), indicating more social interactions on the topic of flood risks and adaptation among those individuals who do not identify as male.

#### 3.2.2 Levels of Intensity of Social Interactions

Using k‐median clustering, we group the respondents based on the intensity of social interactions they engage in when discussing flood risks and adaptation options (Table 2 reports the averages [avg] of different variables that describe these groups). Specifically, besides *Non‐Communicators*, we identify three types of *Communicators* given the three interaction dimensions: *Number of flood conversations, number of people talked to, number of names given in NG*. The *Non‐Communicators* constitute the largest proportion (∼50%) of the survey population. The second‐largest group (40%) are the *Communicators* who interact with 2–4 others on this topic, followed by the *Low‐Communicators* (10%) who only interact with 1–2 others. About 1% of our sample falls under the cluster of *High‐Communicators*, that is, those who ranked high on all three “social interactions” dimensions (e.g., gave at least three names in NG [*number of names given in NG*], discussed floods/adaptation with at least five people last year [*number of flood conversations, number of people talked to*]). Given the small size of this cluster and the number of outliers it contains, we omit it from further analysis and focus on the remaining three groups (Table 2). The differences between the groups are significant in terms of intensity of the social interactions and the risk perception, but not in terms of socio‐demographics (see Appendix D for more details).

**TABLE 2:** Classification of the survey population on the level of intensity of social interactions, with corresponding descriptive statistic on the risk perception and demographic variables. Here, signs * (°) denote variables that differ significantly (not significantly) across the three groups (more information on the tests carried out and the p‐values in Appendix D).

		Non‐Communicators	Low‐Communicators	Communicators
Description		No interactions (NG is 0), majority said they had not talked to anyone/had not had a conversation.	Gave 1–2 names in the name generator, reported having had between 0 and 2 discussions/ people talked to mainly → on average around 1–2 connections	Gave 2–3 names in NG, talked on average to more than 3–4 people/ conversations in last year → on average 2–4 connections
Survey population		∼50%	∼10%	∼40%
Count		786	167	650
Intensity of social interactions	* Avg number of names given in the name generator questions (Number names given in NG)	0	1.14	2.9
	* Avg number of people talked to	0.38	0.41	2.69
	* Avg number of flood conversations	0.62	0.84	3.60
Individual risk perception	* Avg perceived probability	2.30	2.60	3.19
	Proportion of “I don't know” responses for Perceived probability	12%	14%	8%
	* Average worry	1.46	1.68	1.92
	* Fraction with Flood experience	0.20	0.32	0.40
Socio‐demographics	° Income (1–5)	3.28	3.27	3.19
	°Education (1–4)	2.87	2.97	2.90
	* Fraction male	49%	47%	41%
	° Age (1–6)	4.32	4.25	4.28

In general, the correlation between the intensity of the social interactions (number of flood conversations, number of people talked to, number of names given in NG) and the individual risk perceptions (Perceived probability, worry, Fraction with flood experience) is statistically significant across the three groups (see Table D2, Appendix D). It indicates that higher risk perception and interactions on the topic go hand in hand. Notably, a proportion of respondents say “I do not know” in response to the question about the likelihood of flooding at their place. There is no clear trend in relation to the interactions for the “I don't know” responses: The proportion is lowest for the Communicators but increases slightly from the Non‐Communicators to the Low‐Communicators. In terms of demographics (income, education, gender, age), we observe no significant differences among the three groups reporting different interaction levels, apart from the fraction of males differing significantly between the Communicators and Non‐Communicators.

These findings confirm our second hypothesis (H2): Those who communicate with peers on the topic of flooding and adaptation in general have a higher risk perception. Furthermore, the more intense social interactions individuals have on the topic, the higher their risk perception.

### 3.3 Who People Interact With

In total, our sample includes 823 respondents who filled in the questions using the NG approach (i.e. *egos*, see Table E1 in Appendix E for how this data compares to the rest of the survey). The remainder of the respondents did not or could not put down a name of someone they talk to on the topic of flooding, sea‐level rise, climate change, and adaptation. This results in 2100 *alter*—the individuals the respondents described as peers they talk to on the topic—across our three countries. This includes 225 *ego*/564 *alter* pairs from the Dutch case, 336/868 from the UK, and 262/668 from the US, respectively.

We summarize the results of the NG answers in Table 3. Most respondents consider their *alter* to be family and friends. This is by far the most prominent group, with 55% of *alter* classified here. Nearly 15% are neighbors, and 9% of colleagues, who are both sometimes (15%) also considered family and friends. For the remainder (22%), the relation is not clear or unknown. We see some differences between countries: In the US, the proportion of family and friends is the highest among the countries (>60%). The Dutch respondents consider only 50% to be family and friends (see Appendix E for more details on country‐specific differences). Across all relational groups, we do not see large differences in whether the connections are considered to have valuable experience when it comes to adaptation.

**TABLE 3:** Characteristics of people with whom respondents report to engage in social interactions This is based on the data from the Name Generator responses, clustered into four groups of relation and four groups of similarity, aggregated across the respondents from three countries (NL, US, UK).

Who people talk to How similar people are	Family and friends (and no other relation)	Neighbors (that are partly (15%) also family & friends)	Colleagues (that are partly (15%) also family and friends)	Mixed (relation not clear or no clear pattern)	Total over the similarity dimension
Full Homophily (income, age, education, house type)	15%	4%	2%	2%	24%
Age Homophily (all similar age, no similar income)	13%	3%	2%	2%	19%
Location–Education Homophily (some similarities in flood exposure, house type or education, but no similar age or income)	16%	5%	3%	3%	26%
No Homophily	11%	3%	2%	15%	31%
Total per relation category	55%	14%	9%	22%	100%

Looking at the similarity among people engaging in social interactions, we find four relatively evenly split groups. Around 24% fall into the “Full Homophily” cluster, with the *ego* and the characteristics of their *alter* overlapping largely in terms of income, age, education, and house type. Around 19% are just similar in age but not in income (“Age Homophily” cluster), and 26% are dissimilar in both age and income but share similarities in house type, education, or flood exposure (Location–Education Homophily). In the last group, around 31%, the *egos* report having no similarities with their *alter* (No Homophily). Again, the US and NL data diverges the most: US egos only have 22% of their contacts in the “No Homophily” group; for the Dutch respondents, there are 37%. Especially in the cluster on family and friends, for NL, contrary to the US and UK, the less similar, the higher the proportion of Dutch *ego*‐*alter* combinations in that box (Table E2, Appendix E).

Overlapping the similarity and relational data (Table 3), we see that the similarity groups are evenly distributed across the family and friends *alter*. This is also true for the neighbors and colleagues, though the numbers here, in general, are much smaller. In the mixed relation cluster, by far, the largest overlap is with the “no homophily” cluster.

With these findings, our third hypothesis is only partly confirmed: Homophily does play a role in the interactions between individuals. However, homophily across multiple traits is only found for around ¼ of interactions, whereas 1/3 of interactions do not show any traits of homophily. Therefore, H3a is only partly confirmed. The same is true for H3b: Neighbors do play a role in the social interactions of individuals. Nonetheless, they only account for a fraction of these (>15%). Majority of people (55%) engaging in social interactions are family and friends, even if they do not share much on the homophily dimensions.

## 4 Discussion

### 4.1 Social Influence on Risk Perception and Climate Change Adaptation

This study has examined the social influence in relation to risk perception and climate change adaptation in the context of flooding. We use survey data from 1607 respondents from the Netherlands, United Kingdom, and the USA to gain insights into social interactions on this particular topic and to identify what type of people engage in these discussions and to what extent.

Our data shows that the number of people engaging in social interactions about climate risks (like flooding) and adaptation (*N* = 481) as well as the frequency of these interactions (on average less than 1–5 times per year) is moderate if not low, affirming other studies on similar topics (Leiserowitz 2022). This suggests that many individuals do not prioritize the topics of flooding and adaptation high enough to discuss them with their peers, or they lack the network to discuss them with. Our survey was run in areas that have either already experienced floods or are at risk of rising sea levels and intensified flooding. Considering this, we expect that the topic is more prominent among the survey respondents than the rest of the population that does not face these climate‐induced hazards.

We find high correlations between the frequencies of social interactions concerning threat‐related variables being discussed together and coping‐related variables being discussed in concert. This aligns well with theoretical work, for example, with PMT that explicitly distinguishes threat appraisal and coping appraisal as two parts of decision‐making under risk (Grothmann and Reusswig 2006), which is confirmed by our findings. The Dutch data shows one peculiarity: The discussion frequency of the government plans regarding flood preparation and adaptation is highly correlated with the other discussion topics. We do not find this in the UK or US data. This could be due to the special role that public protection against flooding and coastal storms plays in the culture and history of the Netherlands (Wiering and Winnubst 2017). Dutch respondents report high levels of trust in their government and institutions to protect them against flooding and expect them to prioritize protection against this hazard compared to other (climate‐induced) adversities. To conclude, regarding our first research question (RQ1), the intensity of social interactions about climate, flood risks, and private adaptation is low (at least in United Kingdom, the Netherlands, and the USA); these discussions cluster either around threat or coping appraisals as per PMT and are influenced by the wider national adaptation context.

Furthermore, we find that individuals with higher risk perceptions seem to engage more in social interactions with peers on the topic of flood risk and adaptation (RQ2). This correlation does not imply causation: It is not clear if individuals interact more because they perceive climate‐induced risks, like flooding, as higher compared to e.g., *Non‐Communicators* or whether the perceptions of *Communicators* are amplified because they interact more with others who also perceive the risk as high. In either case, social interactions seem to interact with risk perception and play an important role in their formation. They correlate with a heightened risk perception, as outlined in the SARF (Kasperson et al. 2022). The sources of information investigated in this study are the personal experience (flood experience) and the informal social networks where the information is passed on and likely amplified. Of all the factors differentiating between *Communicators* and *Non‐Communicators*, the perceived probability shows the smallest difference, especially among the UK respondents. Many households might be unsure of their flood probability or how it translates to the occurrence of flood events (Strathie et al. 2017). The role of flood experience is prominent in determining who interacts and who does not, highlighting the elevated awareness of individuals with flood experience (Grothmann and Reusswig 2006). Our findings also confirm that female respondents report a higher risk perception (Terpstra and Lindell 2013). When we think about climate action, higher risk perception might, but does not necessarily translate to more action (Wachinger et al. 2013). It may also be dependent on the feelings related to the risk, for example, the climate risk “heat” might be positively connotated in some places and impact the perception (Lefevre et al. 2015). However, the positive correlation between risk perception and interactions indicates that the pair can at least raise awareness. This could be pivotal in triggering transformative change (Wilson et al. 2020).

Our findings on the homophily and neighborhood proportion of the social interactions (RQ3) differ from empirical findings in other contexts; for example, the adoption of solar panels seems to be largely driven by the neighbor effect (Graziano and Gillingham 2015). A common approach in social network and modeling studies in general (Flache et al. 2017; Will et al. 2020) and in the climate change adaptation context in particular (Haer et al. 2016; Yletyinen et al. 2021) is to assume homophily and social influence based on geographical proximity (i.e., among neighbors). Our empirical evidence does not fully support it and finds that the key participants in the discussion of climate change adaptation and risk are friends and family who may but do not necessarily share similar socio‐demographic characteristics or live close by. In summary, in contrast to common assumptions, our findings reveal that neither socio‐demographic homophily nor spatial proximity serves as the defining dimensions for people engaging in social interactions on the topic of climate risks and adaptation. This might be due to the fact that property‐level adaptation measures can take different forms (e.g., flood‐proofing floors or doors), are not as easily translatable from one place to another (e.g., elevation), and in contrast to, for example, private climate mitigation measures, like solar panels, private climate adaptation measures are rarely physically visible to be even noticeable by neighbors (e.g. flood‐proofing sewage pipes or moving a water‐meter upstairs).

The vast majority of social engagements in the context of climate risks, like rising sea levels or flooding, and adaptation considerations, were reported to be informal in our survey. Even though they had such a possibility, our respondents reported no interactions with representatives of institutions or governments concerning climate risks and flood adaptation. Informal interactions with friends, family, colleagues, and neighbors seem to be more relevant. This confirms findings from other research that trust, which is often greater in family and friends compared to governmental institutions, plays an important role in this kind of information‐gathering (Hagen et al. 2016) and capacity building (Adger 2003). The discussion of these topics and the informal information exchange, like via social interactions, can be important factors in building (bonding) social capital and adaptive capacity (Adger 2003; Bixler et al. 2021) and over time driving a shift in social norms (Gelfand et al. 2024). Eventually, a relatively small group might be able to shift the predominant norm (Centola et al. 2018). This can be important to generate tipping points in adaptation uptake and lead to transformational adaptation (Wilson et al. 2020).

### 4.2 Policy Implications

In general, when we dive into the subject of social interactions, we see that climate‐related (flood) risks and adaptation are not at the top of the mind for large parts of the population. The impacts of climate change are becoming more apparent, and individuals need to become more aware. A lack of awareness and of timely adaptation will lead to unnecessary losses and can potentially cost lives (IPCC 2021). Increasing individual awareness and communicating climate risks through information policies might initiate wider engagement on the topic (reach the ∼50% non‐communicators), and may trigger a shift in social norms promoting adaptation actions. Knowledge of the nature of social environment and influence networks can provide gateways for effective channels for amplification of actionable information (Bakshy et al. 2012), and promote social capital that increases individual adaptive capacity (Bixler et al. 2021). This could be done, for example, through targeting specific individuals, providing them with relevant information regarding climate risks and opportunities for adaptation, and leveraging their social influence for spreading that information. Identifying opinion leaders and leveraging their influence is common in other areas like marketing or politics, and there are various ways to detect them (Bamakan et al. 2019). Highly connected or respected individuals could function as “spreading hubs” (e.g., doctor's waiting room, priest, sports coach). Our findings show that these most likely come from within the close circle (∼55% of interactions with family and friends) without specific socio‐demographic characteristics. To activate this channel, the topic of adaptation and climate risks could be brought to places where these people get together, for example, through children's educational institutions, sports events, or concerts. Furthermore, this might be helpful in overcoming a lack of trust in government information (Haynes et al. 2008; Hopp et al. 2020). In addition, targeting specific individuals and supporting them in their adaptation efforts through additional subsidies could help in reaching a critical mass for changing the norms on adaptation. Where this critical mass lies may differ between cultures and places (Gelfand 2021).

Our respondents in the three countries also observe what the government is doing regarding public climate change adaptation with regards to flooding. This is a responsibility but can also provide a chance for governments to lead by good example and provide targeted policies. For example, on the coping side—for example, specific actions that individuals can take to diminish climate‐induced losses for their homes—subsidies and valuable information on flood‐proofing measures might reduce barriers to individual adaptation action. Furthermore, governmental action can increase social interactions on this topic and hence public awareness, which can shift the predominant social norm (Gelfand et al. 2024). Ultimately, activating social norms could trigger positive adaptation tipping points for mass uptake of private action or even support previously unacceptable public government‐led adaptations and eventually reduce damages and costs for immediate disaster relief or long‐term climate costs.

### 4.3 Limitations and Future Work

Gaining insights through the collection of survey data always comes with some limitations. For the US data, only those who retained over the five survey waves answered our social influence questionnaire. These respondents might have a special interest in the topic, are, on average, older than the general population, and have higher levels of education. Furthermore, the role of social influence in driving individual risk perceptions and climate adaptation might be place‐ and time‐specific, especially for areas where individuals ignore or lack hazard experience. At other locations or at different times, the topic might be at the top of everyone's mind, for example, immediately after a flood. People with flood experience are more likely to be involved in social interactions on flooding. We expect that a big flooding event would heavily impact the interaction patterns and, hence, also the survey responses. To account at least for seasonal patterns, we encouraged the respondents to think of social interactions they had both in general and over the last year (e.g., Hurricane Katrina happened in August, as do many other hurricanes in the United States). Furthermore, the survey relies on self‐reporting, and respondents could interpret the questions differently or forget about discussions they had had on the topic, which might influence the responses. As this is a survey run across different countries and cultures, this might also explain some differences we see between the results from the different countries (e.g., who is considered a friend might be different in the United States compared to the Netherlands) (Marin and Hampton 2007). While using the NG approach allows us to gain detailed insights into specific relations, it comes with its own challenges: Limiting the number of names that can be given might influence the responses (Maya Jariego 2018), but this was a deliberate choice to not make the questionnaire too time‐consuming for respondents (Marin and Hampton 2007).

Our study is a starting point for studying social influence in the context of climate risks and adaptation. Future work should investigate the relationship between social influence, risk perceptions, and climate change adaptation in other countries, climate zones, and to other hazards, also taking into account the time‐horizon and perceptions connected to different hazards. Further analysis with a focus on cross‐cultural and cross‐climatic differences can be beneficial and should investigate places that are less studied (e.g., places in a non‐Western context/the Global South). Furthermore, when we think about adaptation, risk perception is only one potential driver of adaptation. Scholars need to better understand how social interactions also influence other adaptation‐related variables and the adaptation actions. Moreover, social interactions (faster) and social norms (slower) change over time and in relation to events (e.g., extreme weather events, political changes). While the patterns elicited can provide an idea of how dynamics might change in relation to changing characteristics of individuals, longitudinal studies could provide more detailed insights into the dynamic nature of social interactions and norms. Through longitudinal data it would allow to understand for example, how the number of people engaged in discussions and the frequency and topics of interactions fluctuate in relation to flood events, and in general, over time. In addition, we do not yet know how interactions in related contexts (e.g., climate policies, discussion of other extreme weather events like heat, politics, comfort of living) lead to spillover effects. To study this, it might be useful to look at the use of different data, for example, from individual engagement with media or social media.

Policies play an important role in initiating and scaling up adaptation action. The role of social influence in relation to the acceptance of adaptation policies and effectiveness of information policies, is not fully clear. Social influence might be an amplifier of information and action, leading even to transformational adaptations (Wilson et al. 2020) or could be one of the constraints leading to adaptation limits (Thomas et al. 2021). Future studies should look into how information policies and social influence can be synergistic in encouraging private climate change adaptation and reducing damages, and how social influence and specifically interactions could be effectively leveraged to overcome soft limits to adaptation (Mechler et al. 2020).

Finally, our findings provide a starting point to further explore the possible evolution of individual risk perceptions or adaptation intentions shaped by social influence. Simulations that account for dynamics of social influences (e.g., networks, agent‐based models) and long‐term longitudinal studies could provide additional insights, possibly about causal directions or which individuals could amplify information effectively and increase the frequency of discussions. They could be used to test effective policies focused on climate change adaptation, but potentially also to explore social tipping and possibilities to use social influence as policy levers for transformational adaptation.

## 5 Conclusions

Social influence, risk perceptions, and climate change adaptation are deeply interconnected. Understanding the role of social influence in this context is crucial for facilitating effective reduction of climate‐induced damages, and for fostering transformational adaptations. Despite the urgent need to better understand whether and how social influence can be a lever for successful climate change adaptation, empirical evidence on its nature is missing.

Our findings offer several novel contributions to the literature. First, even though theories like PMT and, more so, Social Amplification of Risk already acknowledge the role of social influence on climate risk and adaptation perceptions, empirical testing of these impacts is scarce and often does not go beyond registering static effects of descriptive or injunctive social norms. At the same time, models like network analysis and agent‐based modeling already quantify effects of social influence based purely on theory, often overestimating adaptation diffusion and providing overly optimistic policy predictions. Our unique survey data with a focus on flood risk and adaptation from three countries (United Kingdom, United States, the Netherlands; *N* = 1612) with diverse risk profiles permits the elicitation of generalizable trends and patterns. Second, to the best of our knowledge, this is the first dataset reporting these critical details on the understudied and increasingly important topic of social influence in the context of climate change, flood risk, and adaptation perceptions. Third, our findings challenge the common assumptions that adaptation spreads via homophily and within neighborhoods. Fially, our data show that people only sporadically engage in discussion on climate‐induced floods and adaptation to them, despite climate‐related events intensifying globally. These insights can be used to reconstruct networks and allow to model network evolution based on the patterns linked to characteristics of individuals. This provides a basis to quantify the speed and limits of (private) climate change adaptation and risk awareness. Furthermore, they allow to explore when and whether it can ever reach transformational capacity as Wilson and colleagues (2020) suggest, and if positive social tipping points can be triggered in this process. There has been a lot of hope for transformational adaptation to reduce damages if private actors rapidly uptake protective measures to scale up adaptation efforts. It is therefore important to realize that the empirical reality might be different: Despite the rise of climate‐related extremes globally (Delforge et al. 2024; IPCC 2022) and the survey being run in exposed areas, a large number of people (∼50%) do not see them as a priority topic to engage with in their discussions within their own social networks. Nonetheless, those few “communicators” who engage in interactions on flooding and climate change adaptation are important agents of change for the diffusion of information and could help to reach the unengaged. The relation between risk perception and intensity of social interactions indicates that this interplay could become more important as the experience of climate‐related unprecedented extreme events is expected to increase. This will affect more people, and most likely will put these topics higher on the agenda for larger proportions of society. Hopefully, societies could mobilize for a timely (transformational) adaptation before it is put to the test.

## Conflicts of Interest

The authors declare no conflicts of interest.

## Figures and Tables

**FIGURE C1 risa70213-fig-0004:**
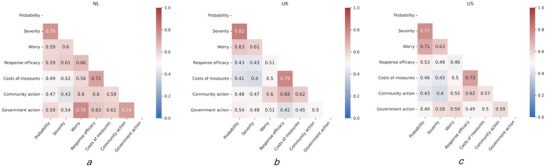
Correlation between the discussion frequency of the different interaction topics for the Netherlands (a), the UK (b), and the USA (c). Here Probability reports how frequently an individual discusses the topic of probability of a climate‐induced hazard with peers. Respectively: Severity—potential severity of a flood event; Worry—how much they worry about a potential flood event; Response efficacy—effectiveness of different adaptation measures; Costs of measures—costs of different adaptation measures; Community action—what actions they can take as a community to adapt; Government action—governments adaptation actions or lack of those.

## Data Availability

The code to replicate this work is at: https://github.com/thoridw and https://github.com/SC3‐TUD

## Endnotes

1
https://yougov.co.uk/

## A. Representativeness of survey population

[Table risa70213-tbl-0004]

**TABLE A1 risa70213-tbl-0004:** Socio‐demographics of the survey population and the distribution of these characteristics over the country.

Explanation			NL		UK		US	
			Survey	Stats^a^ (NL)	Survey	Stats^b^ (England)	Survey	Stats^c^ (US)
Age	1	16–24	10.77%	14.51%	6.61%	11.79%	0.89%	13.20%
	2	25–34	15.79%	15.35%	12.55%	13.50%	5.58%	13.60%
	3	35–44	15.07%	14.22%	15.38%	13.21%	11.16%	13.20%
	4	45–54	17.70%	15.94%	15.25%	12.86%	22.54%	12.20%
	5	55–64	18.18%	16.30%	23.08%	12.67%	28.57%	12.70%
	6	>65	22.49%	23.68%	27.13%	18.61%	31.25%	17.20%
Gender	1	Male	47.37%	49.72%	44.80%	49.00%	46.43%	49.58%
	2	Female	20.33%	50.28%	52.90%	51.00%	53.57%	50.42%
	3,4,5	Other	5.98%		0.8%			
	6	Prefer not to say	26.32%		1.48%			
Income^d^		Avg	37800€	35 400€	29000£	39330£	67000$	105555$
		Median	34500€	31 600€	37400£	32350£	65100$	74755$
		Prefer not to say	64.35%		18.62%		12.95%	
Education	1	No high school	6.70%	28.28%	3.64%	18.20%	0.89%	10.40%
	2	High school	36.12%		33.60%	23.00%	31.25%	45.20%
	3	College degree	42.34%	35.84%	32.66%	22.20%	43.53%	30.40%
	4	Post‐graduate	14.83%	34.19%	27.13%	33.80%	24.33%	14.00%

^a^ Centraal Bureau voor de Statistiek, Netherlands. Data from 2022. Retrieved June 17, 2024.

^b^ Office for National Statistics, United Kingdom. Data from 2022 for England. Retrieved June 17, 2024.

^c^ United States Census Bureau. Data from 2022. Retrieved June 17, 2024.

^d^ Average and median given in income quintiles. Numbers recalculated from income quintile bands.

## B. Descriptive statistics of variables used

[Table risa70213-tbl-0005], [Fig risa70213-fig-0003]

**TABLE B1 risa70213-tbl-0005:** Variables used, the corresponding question, with descriptive statistics.

Question	Variable	Scale	Country	Number answers	Mean	Std	Median	Number “I don't know” responses^a^
*Flood risk related*								
Have you ever personally experienced a flood of any kind?	Flood experience	0: no 1: yes	NL UK US	418 741 448	0.05 0.18 0.50	0.22 0.38 0.50	0 0 1	
How worried or not are you about the potential impact of flooding on your home?	worry	1: Not at all worried – 5: very worried	NL UK US	418 741 448	1.61 1.59 1.93	0.91 0.91 1.00	1 1 2	19 11 0
How often do you think a flood occurs on the property on which you live (e.g. due to rivers or heavy rain, storms and cyclones)?	Perceived probability	1: My house is completely safe 2: Less often than 1 in 500 years 3: Once in 500 years (0.2% chance annually) 4: Once in 200 years (0.5% chance annually) 5: Once in 100 years (1% chance annually) 6: Once in 50 years (2% chance annually) 7: Once in 10 years (10% chance annually) 8: Annually 9: More frequent than once per year	NL UK US	418 741 448	2.47 2.30 3.39	1.81 1.87 2.45	2 1 2	55 61 64
*Interaction related*								
In the past 12 months, approximately how many times have you been involved in a conversation or contributed to a discussion (in person or on social media) involving the subject of flooding?	Number of flood conversations	___ times	NL UK US	418 741 448	1.07 1.89 3.29	5.86 9.89 13.89	0 0 0	
With how many people in your social network (outside your household) do you ever talk about the flood‐related issues (e.g., your concerns about flooding, sea‐level rise or climate change, possible preparations for a flood event, or various adaptation measures against floods for own home)?	Number of people talked to	___ number of people	NL UK US	418 741 448	1.06 1.58 1.85	2.85 7.50 3.99	0 0 0	
*Name Generator questions*								
Please think of three people (outside your household) with whom you discuss flood‐related issues most frequently or whose opinion or experience about the topic matters the most for you. Such flood‐related issues could include concerns about flooding, possible preparations for a flood event, various adaptation measures against floods for own home, sea‐level rise, climate change, etc.								
Please write down the first names of these three people; these could be either their real or fictitious names. It is important that you keep a real person in mind when answering the questions below. If you discuss the flood‐related issues with many people, please feel free to add more names. If you discuss the flood‐related issues only with one or two others, please fill in their names, leaving the rest blank. If you don't discuss this with anyone, just click the arrow to move on.								
Count of names given	Number names given in NG	Name 1: ___ Name 2: ___ Name 3: ___	NL UK US	418 741 448	1.33 1.17 1.50	1.39 1.38 1.39	1 0 1	—
Who is this person to you? Please select all applicable.	Who	Family / friend Neighbor Colleague / business partner Online acquaintance (e.g., though TikTok, Facebook, Instagram, Twitter, etc.) Government official Other	Data from 819 respondents (egos) for in total 2100 alters		0.62 0.17 0.12 0.06 0.05 0.17	0.49 0.38 0.32 0.24 0.22 0.37	1 0 0 0 0 0	
Do you consider yourself to be similar to each of those people in terms of the following characteristics…? Please tick all those you consider to be similar for each person.	similarity	Exposure to floods Type of house Level of education Income Age			0.37 0.34 0.35 0.24 0.33	0.48 0.47 0.48 0.43 0.47	0 0 0 0 0	
*Discussion frequency* How often do you have discussions with other people in your social network about the following flood‐related issues:								
How often floods could affect (or already affect) the area you live in	Discussion frequency probability	1: We discuss it very frequently (at least once per months) 2: We discuss it frequently (more than 5 times per year) 3: We discuss it sometimes (1‐5 times per year) 4: We hardly discuss it (less than once per year) 5: We never discuss it	NL UK US	114 185 182	3.43 3.30 3.15	1.17 0.99 0.97	4 3 3	
How devastating floods could be or are in your area			NL UK US	114 185 182	3.28 3.14 3.21	1.17 0.99 0.97	3 3 3	
How worried you and/or they are about floods			NL UK US	114 185 182	3.28 3.28 3.34	1.30 0.98 1.03	3 4 3	
Possible effectiveness of various flood adaptation measures that people can implement on own home			NL UK US	114 185 182	3.41 3.88 3.74	1.26 1.01 1.01	4 4 4	
Possible costs of various flood adaptation measures that people can implement on own home			NL UK US	114 185 182	3.62 4.00 3.85	1.23 1.06 0.96	4 4 4	
How you could take adaptation measures as a group in your neighborhood or community			NL UK US	114 185 182	3.85 3.94 3.95	1.16 1.11 0.95	4 4 4	
The government's plans or lack of them to deal with flooding in your area			NL UK US	114 185 182	3.61 3.54 3.47	1.26 1.25 1.07	4 4 3.5	

^a^ These responses are not considered when calculating the mean, std, median.

## C. Discussion frequency correlations—per country

[Fig risa70213-fig-0004]

## D. Levels of interactions—differences between the groups

### 1 D1. Distinction between Non‐Communicators, Low Communicators, and Communicators

[Table risa70213-tbl-0006]

**TABLE D1 risa70213-tbl-0006:** Results from the ANOVA analysis between the different communicator groups.

Group 1	Group 2	Variable	Mean difference	*p*‐value	Tukey HSD reject
Communicators	Low Communicators	Number names given in NG Number of people talked to Number of flood conversations Perceived probability Worry Flood experience Fraction Male	−1.7997 −2.2805 −2.7632 −0.6103 −0.2647 −0.0907 0.0588	−1.7997 −2.2805 −2.7632 −0.6103 −0.2647 −0.0907 0.0588	True True True True True False False
Communicators	Non‐Communicators	Number names given in NG Number of people talked to Number of flood conversations Perceived probability Worry Flood experience Fraction Male	−2.9046 −2.3111 −2.9794 −0.8837 −0.4586 −0.1969 0.0961	0.0 0.0 0.0 0.0 0.0 0.0 0.0136	True True True True True True True
Low Communicators	Non‐Communicators	Number names given in NG Number of people talked to Number of flood conversations Perceived probability Worry Flood experience Fraction Male	−1.1497 −0.0306 −0.2162 −0.2734 −0.1939 −0.1062 0.0373	0.0 0.9944 0.9541 0.4644 0.1346 0.0775 0.7736	True False False False False False False

Significant Variables:

– Number names given in NG: ANOVA p‐value = 0.0
– Number of people talked to: ANOVA p‐value = 0.0
– Number of flood conversations: ANOVA p‐value = 0.0
– Perceived probability: ANOVA p‐value = 0.0
– Worry: ANOVA p‐value = 0.0
– Flood experience: ANOVA p‐value = 0.0
– Fraction Male: ANOVA p‐value = 0.0189

Non‐Significant variables:

– Income: ANOVA *p*‐value = 0.6137
– Level of education: ANOVA *p*‐value = 0.7457
– Age: ANOVA *p*‐value = 0.7070

### D2. Correlation between interaction and risk variables

[Table risa70213-tbl-0007]

**TABLE D2 risa70213-tbl-0007:** Spearman's correlation matrix. *p*‐values all <0.05.

	Number names given in NG	Number of people talked to	Number of flood conversations
**Perceived Probability**	0.1458	0.2314	0.2486
**Worry**	0.2096	0.2953	0.3056
**Flood experience**	0.1545	0.2526	0.2900

## E. Who they talk to—per country

[Table risa70213-tbl-0008], [Table risa70213-tbl-0009]

**TABLE E1 risa70213-tbl-0008:** Socio‐demographics of the subset of the survey population that answered the questions using the name generator approach and the distribution of these characteristics over the country.

			NL		UK		US	
		Explanation	Survey	Subset of survey	Survey	Subset of survey	Survey	Subset of survey
**Age**	1	16–24	10.77%	10.76%	6.61%	6.89%	0.89%	0.76%
	2	25–34	15.79%	13.45%	12.55%	14.37%	5.58%	3.44%
	3	35–44	15.07%	12.56%	15.38%	17.37%	11.16%	10.69%
	4	45–54	17.70%	22.42%	15.25%	16.17%	22.54%	23.66%
	5	55–64	18.18%	18.83%	23.08%	21.26%	28.57%	31.30%
	6	>65	22.49%	21.97%	27.13%	23.95%	31.25%	30.15%
**Gender**	1	Male	47.37%	44.84%	44.80%	42.22%	46.43%	41.98%
	2	Female	20.33%	17.49%	52.90%	55.39%	53.57%	58.02%
	3,4,5	Other	5.98%	8.07%	0.8%	0.90%		
	6	Prefer not to say	26.32%	29.60%	1.48%	1.50%		
**Income** ^a^		Avg	37800€	38800€	29000£	29400£	67000$	68000$
		Median	34500€	34500€	37400£	37400£	65100$	67000$
		Prefer not to say	64.35%	58.74%	18.62%	16.47%	12.95%	7.63%
**Education**	1	No high school	6.70%	7.62%	3.64%	3.89%	0.89%	0.76%
	2	High school	36.12%	35.43%	33.60%	30.54%	31.25%	28.63%
	3	College degree	42.34%	42.60%	32.66%	35.93%	43.53%	44.27%
	4	Post‐graduate	14.83%	14.35%	27.13%	26.35%	24.33%	26.34%

^a^ Average and median given in income quintiles. Numbers recalculated from income quintile bands.

**TABLE E2 risa70213-tbl-0009:** Classification of who people talk to overall and per country, linked to other characteristics of the relationship (distance, discussion frequency, experience). This is based on the data from the Name Generator responses, clustered into four groups of relation and four groups of similarity.

Who people talk to How similar people are	Family & Friends (and no other relation)		Neighbors (that are partly (15%) also family & friends)		Colleagues (that are partly (15%) also family and friends)		Mixed (relation not clear or no clear pattern)		Total over the similarity dimension	
Full Homophily (income, age, education, house type)	15%	11%	4%	2%	2%	4%	2%	4%	24%	20%
	19%	15%	6%	4%	2%	2%	1%	2%	27%	23%
Age Homophily (all similar age, no similar income)	13%	9%	3%	2%	2%	1%	2%	3%	19%	15%
	16%	14%	3%	3%	1%	2%	1%	1%	22%	19%
Location‐Education Homophily (some similarities in flood exposure, house type or education, but no similar age or income)	16%	15%	5%	4%	3%	3%	3%	5%	26%	27%
	19%	13%	5%	5%	3%	2%	2%	3%	29%	23%
No Homophily	11%	16%	3%	5%	2%	2%	15%	15%	31%	37%
	7%	11%	1%	2%	2%	3%	12%	17%	22%	34%
Total per relation category	55%	50%	14%	13%	9%	10%	22%	27%	Overall	NL
	61%	54%	15%	15%	7%	8%	17%	22%	US	UK
Valuable experience (M [SD])	2.7 (1.4)		3.1 (1.3)		3.3 (1.4)		3.0 (1.5)		Overall All 1–5 with 1 closest/most frequent discussion/most valuable experience and 5 furthest away/least frequent Discussion/no valuable experience	
Distance (M [SD])	2.8 (1.5)		1.4 (0.9)		3.0 (1.2)		3.0 (1.4)			
Discussion frequency (general) (M [SD])	1.3 (0.8)		1.6 (1.0)		1.5 (1.0)		2.5 (1.4)			
Discussion frequency (on topic) (M [SD])	3.4 (1.3)		3.3 (1.2)		2.9 (1.3)		3.3 (1.4)			
